# Accuracy of diagnosis criteria in patients with suspected diagnosis of sporadic Creutzfeldt-Jakob disease and detection of 14-3-3 protein, France, 1992 to 2009

**DOI:** 10.2807/1560-7917.ES.2017.22.41.16-00715

**Published:** 2017-10-12

**Authors:** Laurene Peckeu, Nicole Delasnerie-Lauprètre, Jean-Philippe Brandel, Dominique Salomon, Véronique Sazdovitch, Jean-Louis Laplanche, Charles Duyckaerts, Danielle Seilhean, Stéphane Haïk, Jean-Jacques Hauw

**Affiliations:** 1 Institut National de la Santé et de la Recherche Médicale (Inserm), U1127, Paris, France; 2 Sorbonne Universités, UPMCUniv Paris 06,UMRS 1127, Paris, France; 3 Institut du cerveau et de la moelle épinière (ICM), Paris, France; 4 Assistance publique–Hôpitaux de Paris (AP-HP), Cellule nationale de référence des maladies de Creutzfeldt-Jakob, Groupe Hospitalier Pitié-Salpêtrière, Paris, France; 5 These authors contributed equally to this study and share first authorship; 6 Institut National de la Santé et de la Recherche Médicale (Inserm), Unité Mixte de Recherche (UMR) 1153, Paris, France; 7 Assistance publique–Hôpitaux de Paris (AP-HP), Laboratoire de Neuropathologie Raymond Escourolle, Groupe Hospitalier Pitié-Salpêtrière, Paris, France; 8 Assistance publique–Hôpitaux de Paris (AP-HP), Service de Biochimie et Biologie Moléculaire, Hôpital Lariboisière; Université Paris Descartes, Paris, France; 9 Académie Nationale de Médecine, Paris, France

**Keywords:** Creutzfeldt-Jakob disease, prion, surveillance, diagnosis criteria, biomarkers

## Abstract

Diagnostic criteria of Creutzfeldt–Jakob disease (CJD), a rare and fatal transmissible nervous system disease with public health implications, are determined by clinical data, electroencephalogram (EEG), detection of 14-3-3 protein in cerebrospinal fluid (CSF), brain magnetic resonance imaging and prion protein gene examination. The specificity of protein 14-3-3 has been questioned. We reviewed data from 1,572 autopsied patients collected over an 18-year period (1992–2009) and assessed whether and how 14-3-3 detection impacted the diagnosis of sporadic CJD in France, and whether this led to the misdiagnosis of treatable disorders. 14-3-3 detection was introduced into diagnostic criteria for CJD in 1998. Diagnostic accuracy decreased from 92% for the 1992–1997 period to 85% for the 1998–2009 period. This was associated with positive detections of 14-3-3 in cases with negative EEG and alternative diagnosis at autopsy. Potentially treatable diseases were found in 163 patients (10.5%). This study confirms the usefulness of the recent modification of diagnosis criteria by the addition of the results of CSF real-time quaking-induced conversion, a method based on prion seed-induced misfolding and aggregation of recombinant prion protein substrate that has proven to be a highly specific test for diagnosis of sporadic CJD.

## Background

Sporadic Creutzfeldt–Jakob disease (sCJD) is the most common form of human prion disease, a group of rapidly fatal untreatable and transmissible encephalopathies. Clinical diagnosis of sCJD has relied on criteria revised over time to incorporate the detection of 14-3-3 protein in the cerebro-spinal fluid (CSF) in 1998 [1] and the results of brain magnetic resonance imaging (MRI) in 2009 [2]. As a marker of acute neuronal injury that does not directly detect abnormal prion propagation, the specificity of 14-3-3 detection has been questioned [3]. This may lead to overdiagnosis of sCJD, and to misdiagnosis of potentially treatable forms of neurological disorders [4]. The introduction of this test into the diagnosis criteria for CJD has considerably impacted the organisation of CJD surveillance in France. Since 1998, patient referral for autopsy to the French National Network for CJD surveillance has relied mainly on the report of 14-3-3 detection performed in five affiliated laboratories.

In a series of 1,572 autopsied patients collected over an 18-year period, we assessed whether there was overdiagnosis of sCJD in France and how the 14-3-3 detection impacted on this, and could lead to the misdiagnosis of treatable disorders.

## Methods

### Cohort study

We reviewed clinical, laboratory, electroencephalographic (EEG) and pathological data for 1,572 patients who were referred to the French neuropathology CJD network for autopsy between 1992 and 2009, and were included in the CJD National Reference Centre register [5-11].

### Diagnostic assessment

Data provided by physicians to the CJD National Reference Centre register and to the neuropathologists for autopsy request were analysed (ND-L, J-PB, J-JH), distinguishing between possible and probable cases according to the World Health Organization (WHO) criteria (Table 1). For EEG data (available for 1,266 cases: 888 sCJD and 378 non-CJD), periodic (or pseudo-periodic) sharp wave complexes considered to be typical of CJD by the referring physicians, were recorded (EEGs were not systematically reviewed). CSF 14-3-3 protein immunoreactivity was available in 1,123 cases (749 sCJD and 374 other diagnoses). Analysis for the prion protein gene (*PRNP*) was performed in 871 cases (705 sCJD and 166 non-CJD).

**Table 1:** World Health Organization criteria for sporadic Creutzfeldt-Jakob disease, 1992–2009

1992–1998			1999–2009		
**- Definite CJD: neuropathological diagnosis** **- Probable CJD: I + 2 from column II + III** **- Possible CJD: I + 2 from column II + disease duration < 2 years**			**- Definite CJD: neuropathological diagnosis** **- Probable CJD: I + 2 from column II + III, or possible CJD + CSF 14-3-3 detection** **- Possible CJD: I + 2 from column II + disease duration < 2 years**		
**I**		Rapidly progressive dementia	**I**		Rapidly progressive dementia
II	A	Myoclonus	II	A	Myoclonus
	B	Cerebellar or visual signs		B	Cerebellar or visual signs
	C	Pyramidal or extrapyramidal signs		C	Pyramidal or extrapyramidal signs
	D	Akinetic mutism		D	Akinetic mutism
III		Typical EEG	III		Typical EEG

CJD: Creutzfeldt-Jakob disease; CSF: cerebrospinal fluid; EEG: electroencephalogram.

### Prion protein gene, Western blotting and neuropathological studies

*PRNP* analysis was performed as described [5,6] when informed consent was obtained from either the patient or the next of kin. Western blotting and neuropathology were performed following established methods [8-10]: after gross examination, one hemi-brain (left or right at random) was frozen at autopsy for PrPsc displaying (Western blot), the other fixed in formalin for 1 month. Hemi-brains were kept in the brain bank. At least eight formalin-fixed samples included: (i) transverse sections of cerebral cortex (middle frontal and superior temporal gyri, medial temporal gyrus including hippocampal formation and parahippocampal region, and cuneus); (ii) transverse sections of basal ganglia (head of caudate nucleus and anterior putamen, thalamic centromedian nucleus and mammillary bodies); (iii) sections perpendicular to the axis of the brains stem (cerebellar vermis including the nucleus dentatus; brain stem including the substantia nigra). After partial inactivation by 96% formic acid for 1 hour and paraffin embedding, 4 μm-thick sections were stained with haematein-eosin and periodic acid-Schiff techniques. Since 1998, a minimum of two sections (cerebral and cerebellar cortices) have been immunolabelled with PrP antibodies, using 12F10 monoclonal antibody (Bertin, Montigny le Bretonneux, France) [10]. Each specimen was examined by at least two neuropathologists (VS, DS, CD, J-JH), and, in most cases, neuropathologists from other centres of the French neuropathology network of Creutzfeldt-Jakob disease). Combined disorders were classified considering both therapeutic possibilities and prion diseases unique infection control problem. For example, a case with lesions of Wernicke’s encephalopathy associated with a few Alzheimer lesions was classified as encephalopathy. However, when lesions of Wernicke’s encephalopathy were associated with CJD, the case was classified as CJD*.*

### Ethics

No autopsy can be performed in France unless the patient’s next of kin can testify that the deceased did not object to autopsy. The national computerised registry of objection to autopsy is systematically consulted. Another specific consent is needed for genetic studies. The Commission Nationale de l’Informatique et des Libertés allows the surveillance register to publish anonymised data [11].

### Statistics

Statistical analysis was performed using STATA 12.1 (StataCorp. 2011. Stata Statistical Software: Release 12. College Station, Texas: StataCorp LP). Data are described as mean (standard deviation) and percentage, and compared with means comparison test and Fisher’s exact test. A p value ≤ 0.05 was considered to be statistically significant.

## Results

**Figure 1:**
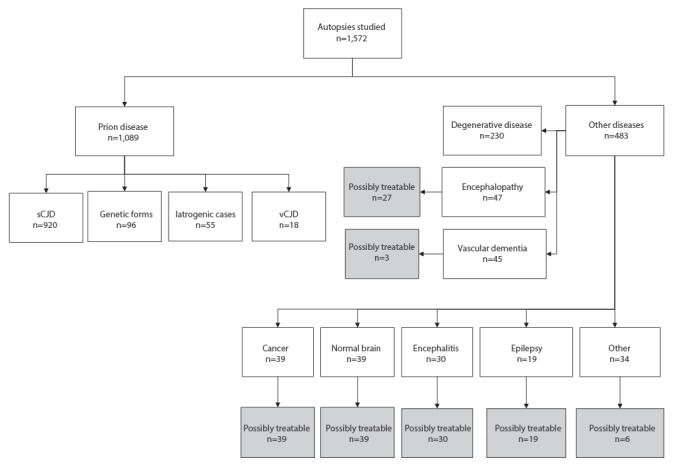
Number of patients with autopsy-proven prion diseases and alternative diagnoses, including possibly treatable diseases, France, 1992–2009

Data from 1,572 autopsies, performed between January 1992 and December 2009, were studied. This corresponds to 11.8% of the 13,384 patients referred to the CJD surveillance network. Excluding genetic (n=96), iatrogenic (n=55), and variant CJD (vCJD) (n=18), there were 920 sCJD cases and 483 cases of non-prion diseases (Figure 1).

sCJD: sporadic Creutzfeldt-Jakob disease; vCJD: variant Creutzfeldt-Jakob disease.

Among these cases there were 230 patients with neurodegenerative diseases (47.6%). As detailed in Table 2, these were mainly Alzheimer’s disease (74.8%), Lewy body dementia and Parkinson’s disease (14.8%). and fronto-temporal lobar degeneration (5.7%). There were 47 cases of non-prion anoxic/metabolic/toxic encephalopathy (9.7%), 45 cases of vascular dementia (9.3%), 39 cases of cancer-related disorder (8.1%), 30 cases of encephalitis (6.2%) and 19 cases of non-convulsive status epilepticus with no cause found at autopsy (3.9%). In 39 patients, no history of dementia and no histopathological lesions were found and we categorised them as ‘normal brain’. In 28 patients, there was a history of dementia and no typical histopathological lesions were found. We categorised them as ‘unspecified dementia after clinicopathological correlation’ (Table 2). A total of 163 patients with potentially treatable diseases had been suspected of having CJD. Of these, 36 patients fulfilled criteria for probable sCJD (Table 3).

**Table 2:** Alternative diagnoses in autopsied patients referred to the national Creutzfeldt-Jakob disease surveillance network, France, 1992–2009 (n=483)

Classification of disease	Alternative diagnosis	n
**Degenerative diseases** **(n = 230)**	Alzheimer's disease	172
	Lewy body dementia and Parkinson’s disease	34
	Frontotemporal lobar degeneration	13
	Amyotrophic lateral sclerosis and frontotemporal dementia	7
	Olivopontocerebellar atrophy	2
	Argyrophylic grain disease	1
	Unspecified degenerative disease	1
**Encephalopathies** (n = 47)	Metabolic encephalopathy	17
	Anoxic encephalopathy	15
	Vitamin B deficiency	13
	Unspecified encephalopathy	2
**Vascular dementia** (n = 45)	Multi-infarct and/or microangiopathy dementia	35
	Mixed dementia	7
	Horton disease/ Wegener disease	2
	Unspecified vasculitis	1
**Cancer** (n = 39)	Primary intra-cranial tumour	26
	Intracerebral metastases	4
	Paraneoplastic encephalitis	7
	Leukoencephalopathy with thymoma	1
	Radiotherapy related grade I astrocytoma	1
**Normal brain** (n = 39)	NA	39
**Others** (n = 34)	Unspecified dementia after clinicopathological correlation	28
	Heterotopia	2
	Multiple sclerosis	2
	Malignant neuroleptic syndrome	1
	Cavernoma of brain stem	1
**Encephalitis** (n = 30)	Viral encephalitis	11
	Unspecified encephalitis	5
	Meningoencephalitis	9
	Fungal infection (candidiasis or cryptococcosis)	2
	Rasmussen's encephalitis	3
**Status epilepticus** (n = 19)	NA	19
**Total**		**483**

NA: not applicable.

**Table 3:** Ante-mortem classification of patients with potentially treatable diseases and definite diagnosis in patients with a diagnosis of probable sporadic Creutzfeldt-Jakob disease before and after introduction of 14-3-3 detection, France, 1992–2009

Period of time	Ante-mortem classification of patients with potentially treatable diseases					Definite diagnosis among patients with a probable diagnosis of sCJD				
	Probable sCJD		Possible sCJD		Total	sCJD		Other diagnosis		Total
1992–1997	1	4%	21	96%	**22**	151	92%	13	8%	**164**
1998–2009	35	26%	102	74%	**137**	586	85%	100	15%	**686**
**Total**	**36**	**23%**	**123**	**77%**	**159**	**737**	**87%**	**113**	**13%**	**850**

sCJD: sporadic Creutzfeldt-Jakob disease.

### Impact of 14-3-3 detection on the activity of surveillance network

**Figure 2:**
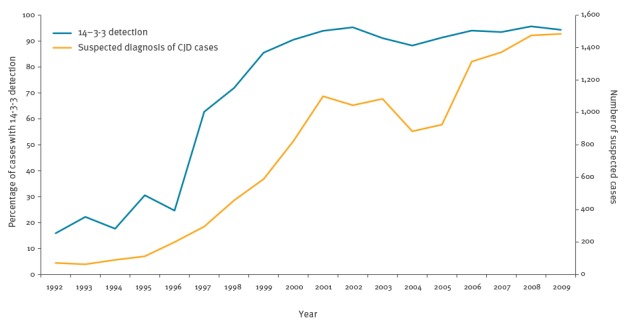
Evolution of the number of suspected cases of Creutzfeldt-Jakob disease and of the percentage of cases with 14-3-3 detection, France,1992–2009

In 1992 14-3-3 detection was performed in under 20% of patients referred to the French neuropathology CJD network for autopsy, but by 1999 it was performed for 85% of such patients, and for over 90% after 2000 (Figure 2). Simultaneously, surveillance was reorganised to integrate this test: all patients in whom 14-3-3 was detected were referred to the French network and their diagnostic status was systematically analysed according to the WHO criteria (Table 1). This was associated with an important increase of the number of suspected cases between 1997 and 2001 (Figure 2).

CJD: Creutzfeldt-Jakob disease

### Accuracy of diagnosis criteria in suspected Creutzfeldt-Jakob disease cases and detection of 14-3-3 protein

From January 1992 to December 1997, the proportion of definite CJD among patients classified as probable CJD (diagnosis accuracy) was 92%. This proportion significantly decreased to 85% from January 1998 to December 2009 (p = 0.014) (Table 3). This was due to the detection of 14-3-3 protein in cases with negative EEG and alternative diagnosis at autopsy. Among 686 autopsied patients classified as probable CJD, 36 were false probable CJD cases due to CSF 14-3-3 detection only (Table 4).

**Table 4:** Alternative diagnoses of treatable diseases in autopsied patients classified as probable sporadic CJD while alive on account of 14-3-3 detection only, France, 1992–2009 (n=36)

Alternative diagnosis		n
**Encephalopathies** (n = 6)	Metabolic encephalopathy^a^	3
	Vitamin B deficiency	3
**Vascular dementia** (n = 1)	Unspecified vasculitis	1
**Cancer** (n = 12)	Primary Intracranial tumour	9
	Paraneoplastic encephalitis	2
	Leukoencephalopathy with thymoma	1
**Normal brain** (n = 6)	NA	6
**Others** (n = 1)	Malignant neuroleptic syndrome	1
**Encephalitis** (n = 7)	Viral encephalitis	3
	Unspecified encephalitis	2
	Meningoencephalitis	1
	Rasmussen's encephalitis	1
**Status epilepticus** n = 3	NA	3
**Total**		**36**

^a^ One patient with a metabolic encephalopathy was the only probable CJD case in the period 1992–1997.

CJD: Creutzfeldt-Jakob disease; NA: not applicable.

### Alternative diagnoses found in patients with probable sCJD and detection of 14-3-3 protein

Since the introduction of 14-3-3 detection in 1998, the present study reveals a decrease in the proportion of neurodegenerative diseases and an increase of the proportion of cancers, vascular diseases, encephalitides and epilepsy among patients with an alternative diagnosis (Table 5). In those patients with non-neurodegenerative diseases, 14-3-3 was detected in 50 cases (44%) of the total of 114 cases. In neurodegenerative diseases, 49 (31%) of the total of 160 patients had positive 14-3-3 detection.

**Table 5:** Evolution of alternative diagnoses for sporadic Creutzfeldt-Jakob disease, France, 1992–2009 (n=483)

Non-CJD cases	1992–1997		1998–2009		Total
	n	%	n	%	
Degenerative diseases	39	53.4	191	46.6	230
Encephalopathies	7	9.6	40	9.8	47
Cancer	2	2.7	37	9.0	39
Vascular diseases	4	5.5	41	10.0	45
Normal brain	11	15.1	28	6.8	39
Encephalitides	2	2.7	28	6.8	30
Epilepsy	1	1.4	18	4.4	19
Others	7	9.6	27	6.6	34
Total	73	100	410	100	483

CJD: Creutzfeldt-Jakob disease.

### Potentially treatable diseases with positive detection of 14-3-3 protein

Possibly treatable cases (163, 33.7% of non-CJD cases) included 39 cancer-related disorders, 27 encephalopathies (Wernicke’s encephalopathy or pellagra), 30 encephalitides (some viral diseases, such as subacute sclerosing panencephalitis, have poor outcomes, but may benefit from therapeutic trials), 39 normal brains (e.g. unrecognised delirium, drug abuse, withdrawal syndrome, psychiatric disease), 19 non-convulsive status epilepticus with no cause found at autopsy and a number of other disorders (Figure 1). Interestingly, with the introduction of 14-3-3 detection, the number of patients classified as probable sCJD with a final diagnosis of potentially treatable diseases significantly increased from 1 to 35 (p = 0.01) (Table 3). Among these 35 patients, 26 showed positive detection of 14-3-3 and 17 were classified as probable sCJD according to positive 14-3-3 only without periodic sharp wave complexes on EEG.

The mean age at death in sCJD and non-CJD cases were similar: (age +/ − standard deviation (SD) = 68.7 years +/ − 9.5, vs 68.9 years +/ − 14.7). However, before the age of 50 and after the age of 80 years, misdiagnoses were more numerous than between 50 and 80 years (p < 0.0001). Regarding specifically treatable diseases, 68% (111/163) of the cases were aged 60 years and older.

The distribution of the *PRNP* genotypes at codon 129 in 705 sCJD and 166 non-CJD cases revealed significant differences. Among sCJD patients, the genotype was: Met-Met (421/705) (59.7%), Met-Val (139/705) (19.7%), Val-Val (145/705) (20.6%). Among the other cases, it was Met-Met (75/166) (45.2%), Met-Val (73/166) (44.0%), Val-Val (18/166) (10.8%); (p < 0.002).

## Discussion

As compared with the other European countries belonging to the EuroCJD network, France shows one of the highest number of 14-3-3 protein referrals. This largely contributed to the high incidence of sCJD in France and is regarded as a marker of high surveillance intensity [12]. An intensive surveillance fits well with the primary aim of the EuroCJD network which is to identify vCJD cases among the CJD population. However, while the highest sensitivity of diagnosis criteria is commendable, the present study shows that the introduction of 14-3-3 detection in the definition of probable cases coincided with a loss of specificity leading to the misdiagnosis of potentially treatable diseases. Without 14-3-3, the diagnostic sensitivity in probable cases was 56.1% and the specificity 95.6%. With 14-3-3 detection, the sensitivity was 82.4%, and the specificity 75.6%.

Other factors may be responsible for the high number of misdiagnoses. In all European countries, special attention was paid to CJD from March 1996 onwards, when vCJD was first reported in the United Kingdom [13], introducing a public health motivation for diagnosis coinciding with the introduction of CSF 14-3-3 in the diagnostic criteria. Specific to our research, another possible bias may be the study of autopsy cases only. Medical staff may ask for an autopsy to be performed because there is doubt about the CJD diagnosis. As a consequence, other diagnosistic alternatives may have been discussed before the patient’s death without formal report to the neuropathologist. Also, it may be pointed out that in France, in case of suspicion of CJD, any medical device that has come into contact with the patient should be destroyed unless autopsy dismisses the diagnosis. An increase in the use of CSF 14-3-3 and in the percentage of probable sCJD solely with positive CSF 14-3-3 is not specific to France, however, as shown by the study of national databases of 11 members of EUROCJD-Consortium, including nine European countries, Australia and Canada during the period 1993–2002 [14].

The 14-3-3 proteins are a group of 30-kDa proteins involved in signal transduction and apoptosis and are normally expressed by brain neurons. Their detection in CSF is regarded as a marker of subacute neuronal suffering. It thus can be observed in various neurological diseases including epilepsy, cancer, and paraneoplastic encephalitis. In our series, during the 1998–2009 period, when performed, 14-3-3 protein was detected in 15/18 cases of epilepsy, 15/37 cases of cancer and 7/28 cases with encephalitis, all potentially treatable diseases. Because of its lack of specificity, interpretation of 14-3-3 detection results must be performed within the context of the patient's clinical history and must not result in potentially useful therapies being dismissed. Importantly, a positive 14-3-3 may be drug-induced [15], which could explain some of the ‘normal brains’ found at autopsy.

The potentially treatable alternative diagnoses in France are similar to those of other national series reported in Europe [14,16-19] and in various other countries: the United States [20,21], Japan [22], Argentina [23] and China [24]. In the Dutch series that included suspicions between 1998 and 2009, a substantial group (ca 14.0%) suffered from potentially treatable disorders such as infectious disease, cancer-associated disease, or toxic/metabolic disorders. The authors pointed out that many of these patients were referred to the Netherlands National Prion Disease Registry based on a positive 14-3-3 test in cerebrospinal fluid [19]. We confirm these data in the present large series: the frequency of the most common potentially treatable alternative diagnoses increased with the introduction of 14-3-3 protein detection, to reach values like those observed in the Dutch study.

In 2009, the presence of high signals of the striatum on T2-weighted sequences and diffusion-weighted imaging in brain MRI was introduced among the major criteria of probable sCJD [2]. It is too early to assess the impact of this improvement on the accuracy of these criteria. However, since these MRI alterations are rarely observed in most of the treatable alternative diagnoses, an increase in specificity should be expected.

Among the various researches for newer, more predictive, and informative biomarkers [[ 25], a direct method for the diagnostic of sCJD that detects the seeding activity of abnormal PrP assemblies in the CSF has been proposed [26]. Real-time quaking-induced conversion (RT-QuIC) showed a specificity of 99 to 100% and a sensitivity similar to that of 14-3-3 detection [27,28]. Its validation using large series in European countries is pending. According to the results and to the reliability and the robustness of the method [27], RT-QuIC results in CSF or other tissues were recently added to the diagnosis criteria for sCJD by the EuroCJD consortium [28,29]. In the event that a specificity of 100% should be confirmed, especially in series including treatable alternative diagnoses, this method should contribute to considerably improve the diagnosis of CJD and other disorders leading to rapidly progressive dementia, including treatable diseases.
